# A Substantive Narrative Review on the Usage of Lidocaine in Cataract Surgery

**DOI:** 10.7759/cureus.19138

**Published:** 2021-10-30

**Authors:** Akshay J Reddy, Allen Dang, Amy A Dao, Gordon Arakji, Joshua Cherian, Hetal Brahmbhatt

**Affiliations:** 1 Opthalmology, California Northstate University College of Medicine, Elk Grove, USA; 2 Anaesthesia, California Northstate University College of Medicine, Elk Grove, USA; 3 Health Sciences, California Northstate University College of Health Sciences, Rancho Cordova, USA; 4 Psychiatry, Mercy General Hospital, Sacramento, USA

**Keywords:** fentanyl, corneal epithelium, phenylephrine, lidocaine, cataract

## Abstract

Cataracts are a disease that causes a gradual decrease in visual prowess and requires surgery when the symptoms progress to an abhorrent state. This disease can be treated through surgical procedures that use anesthetics, such as lidocaine. Through inhibiting sensory nerve propagation to the brain, lidocaine plays an invaluable part in reducing pain for patients that undergo cataract surgery. Current clinical practice commonly utilizes 2% lidocaine with fentanyl as a combination agent. However, recent studies have reported that concentrations higher than 1% can cause substantial alteration to corneal epithelium cells. Additionally, fentanyl is cited as an extremely addictive opioid inappropriate for continual use in cataract surgeries. In this review, the authors examine the application and concentration of lidocaine, along with the various combination agents that were reported in several studies that describe the usage of the anesthetic during cataract surgery. Within the review, it was found that most surgeons generally only use lidocaine gel on the corneal epithelium tissue of patients during cataract surgery. Perhaps this standard could change over time as it is generally known that using intracameral injections in conjunction with topical anesthesia produces better patient outcomes. The authors find that although anesthetics and surgical treatment for cataracts are generally beneficial for patients, there are still many adjustments that could be implemented to enhance patient outcomes.

## Introduction and background

A cataract is an ocular disease that occurs when the lens of a patient’s eye becomes cloudy. Cataracts generally occur as a result of the aging process. In fact, it is estimated that one out of every five individuals from the ages of 65 - 74 has a cataract [1]. It is theorized that due to ultraviolet (UV) light exposure over time, the collagen within the eye’s lens deteriorates which causes the normally clear lens to become cloudy in appearance. In fact, one study has shown that high exposure to UV light can cause a 60% increase in the risk of developing cataracts [2]. This deterioration caused by UV light can lead to hazy vision and lower perception during the night. Cataracts generally cause a gradual decrease in vision over time but don’t cause physical pain. Although cataracts are a treatable disease, they are also the leading cause of blindness within the developing world due to a lack of access to surgery. Cataracts can be surgically removed from a patient and replaced with an intraocular lens (IOL). It is estimated that over 10 million cataract surgeries are performed each year around the world [3]. In order to diminish the pain that patients may experience during an operation, it is quite common that surgeons use anesthetics when performing this procedure. One of the more popular anesthetics for this procedure is lidocaine. Due to the fact that this anesthetic is quite commonly used for this procedure, it is important to outline and understand the proper standards and protocols that are necessary when utilizing this medication in order to protect and maintain patient health. The purpose of this review is to analyze the current usage and application of lidocaine on patients during cataract surgery. 

## Review

Cataract surgery

One of the first steps when performing cataract surgery is to apply topical anesthesia to a patient's eye. This is done to ensure that minimal harm will come to the patient during surgery. Physicians tend to utilize lidocaine as the primary topical anesthetic for this surgery. As the most widely used local anesthetic by almost all medical specialties, lidocaine has proven to be a game-changing aspect of surgical and invasive procedures [4]. Through its inhibition of sensory nerve endings, lidocaine prevents pain signals from reaching the brain and effectively works to reduce pain felt by patients [5]. Without anesthetics, patients would experience a severe amount of pain during cataract surgery. Therefore, when performing cataract surgery, it is important to ensure that the right type of anesthetics is being used to minimize patient harm. With the cataract surgical rate increasing each year and the need for efficient and simplified preoperative preparation, the use of topical lidocaine has significantly increased within the clinics of ophthalmologists [6]. In a study performed at the University of Berlin, the use of lidocaine for cataract surgery was shown to be effective and non-harmful, given that doses were administered at 1% [7]. Using corneas excised from pig eyes that were enucleated, the group tested the effects of unpreserved lidocaine hydrochloride at 1%, 5%, and 10% and found that substantial corneal endothelial alteration was found with uses of lidocaine 5% and complete endothelial necrosis was found with the use of lidocaine at 10% [7]. Their results came as a surprise, given that so many clinics use 2% as their standard dose, and thus presents a point of investigation in our paper. Furthermore, in a study conducted at the University of Florida, the effect of combination agents, phenylephrine and ketorolac 1.0%/0.3% (alongside lidocaine 1%), were tested to evaluate its possible synergistic effects with lidocaine in 60 patients who underwent femtosecond laser-assisted cataract surgery [8]. They found that, compared to the use of epinephrine with lidocaine 1% as the control, the experimental group that was treated with phenylephrine and ketorolac 1.0%/0.3%, along with lidocaine 1%, had a decrease in the likelihood that patients experienced moderate-to-severe pain or requested for fentanyl for additional pain relief. Their results pose another area of investigation that we kept in mind as we cross-examined multiple articles and their use of different combination agents with lidocaine [8].

Combination agents

Combination agents play a key role in maintaining the analgesic effects of specific drugs. Therefore, it is important to analyze the different combination agents that can be used with lidocaine to enhance its analgesic effects. After an analysis of the review articles seen in Table 1, it was found that phenylephrine, tropicamide, tetracaine, povidone-iodine, and fentanyl were the most commonly used combination agents with lidocaine in cataract surgeries [5-6, 8-33]. It is unsurprising to see phenylephrine, tropicamide, and tetracaine as three of the most popular combination agents since they are used in conjunction as an intracameral preparation to achieve sustained mydriasis throughout ophthalmic surgeries [34]. Necessary pupillary dilation is required in cataract surgeries as surgeons require clear views of the structures behind the iris to prevent complications to the patient, such as bleeding, anterior capsule damage, and misalignment of the IOL (Obi AA, Penwarden A, Niskopolou M, Pool T: abstract 749 - Minimum eyedrop and intracameral pupil dilation for cataract surgery. Annual Meeting of the Assn. for Research in Vision and Ophthalmology, Fort Lauderdale, FL, May 1-4, 2005. http://iovs.arvojournals.org/article.aspx?articleid=2400485). On the other hand, the antiseptic povidone-iodine was utilized in three different articles. This is to be expected as the most common complication post-cataract surgery is bacterial infections resulting in postoperative endophthalmitis. The utilization of povidone-iodine has been seen to reduce the incidence of postoperative infections in cataract surgery [35]. The narcotic fentanyl was also seen in three distinct articles as an anesthetic to provide patient comfort during cataract operations. However, this practice is widely alarming as fentanyl is a highly addictive opioid that is 50 to 100 times stronger than morphine [36]. Current standards should be reevaluated, and the high usage of fentanyl should be reconsidered. Within this article review, over 25 unique combination agents were used in conjunction with varying techniques of lidocaine usage. Patients undergoing cataract surgeries are generally the elderly population [37]. The employment of exuberant amounts of combination agents accounts for underlying health concerns and the individual needs of each patient.

**Table 1:** An Analysis of the Application of Lidocaine and Other Combination Agents in Cataract Surgery

Author (Year)	Concentration (Lidocaine)	Combination Agents	Area(s) of Applied Anesthetic	Sample size (Patients)
Aguliar (2021) [9]	2%	1% phenylephrine	Corneal epithelium	70
Assia (1999) [10]	2%	2% esracain	Corneal epithelium	100
Auclin (2005) [11]	2%	Not applicable	Corneal epithelium	600
Bardocci (2003) [12]	2%, 4%	Tropicamide, diazepam	Corneal epithelium	118
Barequet (1999) [13]	2% gel	0.5% tetracaine	Conjunctival fornices	25
Bournas (2005) [14]	1%	1.5% sodium hyaluronate	Corneal epithelium	437
Busbee (2010) [15]	1.5%, 2.5%, 3.5%	Not applicable	Corneal epithelium, conjunctival	209
Chalam (2009) [16]	2%	0.5% tetracaine	Corneal epithelium	122
Chandra (2018) [17]	2%	0.5% tetracaine	Corneal epithelium	72
Chung (2004) [18]	2%	0.5% tropicamide	Corneal epithelium	76
Donnenfeld (2019) [8]	1%	Phenylephrine and ketorolac 1.0%/0.3% vs. epinephrine (1 mg/ mL)	Intracameral injection	60
Fernandes (2013) [19]	2%	Proxymetacaine 5%, povidone-iodine 5%	Corneal epithelium	106
Fraunfelder (2009) [5]	1.5%, 2%, 2.5%, 3.5%	5% povidone-iodine	Corneal epithelium	26
Gilani (2014) [20]	2%	Fentanyl, atracurium, propofol	Intravenous	240
Jinapriya (2012) [6]	2%	Phenylephrine 3.4%, tropicamide 0.34%, and diclofenac 0.016%	Corneal epithelium	20
Kirber (2000) [21]	2%	Methylprednisolone acetate, triamcinolone acetonide	Corneal epithelium	3
Kwok (2006) [22]	2%	2.5% phenylephrine, 1% cyclopentolate	Corneal epithelium	41
Miller (2005) [23]	2%	Not applicable	Corneal epithelium, subconjunctival injection	7
Page (2009) [24]	1.5%, 2%, 2.5%, 3.5%	5% povidone-iodine	Corneal epithelium	234
Pham (2010) [25]	1%	Oxybutynin	Corneal epithelium	1010
Santiago (2014) [26]	2%	Clonidine	Inferior conjunctival sac	40
Sinha (2009) [27]	2%	Isoflurane propofol oxygen in nitrous oxide, ketorolac fentanyl	Corneal epithelium	100
Soliman (2004) [28]	2%	0.5% bupivacaine, 0.4% benoxinate	Corneal epithelium, sub-Tenon’s supplemental injection	90
Theocharis (2007) [29]	1%	Morphine dixyrazine	Corneal epithelium (jelly in conjunctival fornices)	69
Thill (2005) [30]	2%, 1%	0.5% bupivacaine, 1% diclofenac, 1% oxybuprocaine	Corneal epithelium, intracameral injection	33
Tsoumani (2010) [31]	2%	0.5% tetracaine	Corneal epithelium	51
Venkatakrishnan (2011) [32]	2%	0.015 mg/kg intravenous midazolam	Corneal epithelium, conjunctiva	78
Vonjagow (2007) [33]	2%, 1%	Not applicable	Corneal epithelium, intracameral injection	45

Application of anesthesia

When performing cataract surgery, it is not only important to ensure that the right type of anesthetic is being used, it is quintessential to ensure that the anesthetic is placed on the appropriate area. Even if physicians use the right type of anesthetic, if they do not use the correct application of the drug, the patient will feel an immense amount of pain during surgery. Therefore, understanding the application of anesthesia is crucial when trying to improve patient care for individuals who undergo cataract surgery. Based on the data that was reported in Table 1, the most common area of application for lidocaine is the corneal epithelium [5-6, 8-33]. This is most likely due to the fact that during cataract surgery, lidocaine is given to the patient in gel form. The gel is applied topically on the patient's eye but gets absorbed by the corneal tissue. The medication is then able to spread throughout the orbit and produce an analgesic effect. Another reason for this could be because applying anesthesia to the corneal epithelium is a non-invasive application of the drug which leads to less patient complications. There were a few reported studies where lidocaine was not directly applied on the cornea but rather injected into the intracameral region of patients’ eyes. This could potentially have been since when intracameral injections of anesthetics are used, the patient can experience a faster rate of visual rehabilitation and a decrease in their room turnover time [38]. It may be favorable for physicians to consider utilizing intracameral injections along with topical anesthetics to ensure that the patient receives the best possible outcome. However, physicians should also ensure that invasive applications of anesthetics are only done when necessary and that the concentration of anesthetic used does not cause physical harm to the patients. 

Concentration

**Figure 1:**
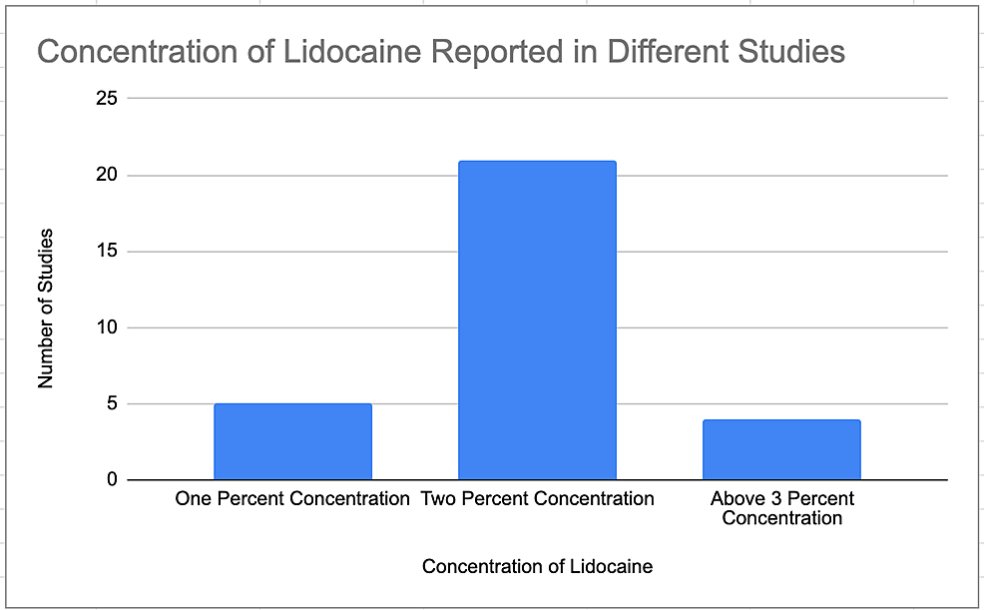
Lidocaine Concentration Distribution of the Studies Within the Literature Review

The concentration of lidocaine being used during cataract surgery can vary depending upon a multitude of factors including patient complications, number of drops, or even clinical regulations. Within the review, it was found that the most common concentration of lidocaine that was used was at 2% [5-6, 8-33]. In fact, according to the data presented in Figure 1, 21 of the studies within the review state that the concentration of lidocaine that was used for the procedure was 2%. This most likely indicates that this concentration is the bare minimum that needs to be utilized in order to achieve an analgesic effect within the patients. Although the majority of the studies used a concentration of 2% when treating patients with lidocaine, there were a few studies within the review that utilized lidocaine at concentrations of 1% and 1.5% [5, 8, 14-15, 24-25, 29-30, 33]. This could potentially indicate that different patients have different thresholds to achieve the analgesic effect or that different procedures with variable combination agents require a lower concentration of lidocaine to ensure the safety of the patient. There were also a few articles within the review that utilized lidocaine at concentrations of 2.5%, 3.5%, and 4% [5, 12, 15, 24]. This demonstrates that there may be a need to increase the amount of anesthetic that is used on patients to ensure that they are placed in an analgesic state during surgery. Additionally, this may also indicate that a higher concentration of the anesthetic is needed for cataract surgeries that are more invasive. The increase in the standard concentration of 1% may indicate that specific protocol is not being followed or that the standard of care may need to be changed [7]. It is quintessential for physicians to keep this in mind in order to minimize the pain that patients feel when they are undergoing surgical treatment. 

A plethora of applications can improve future cataract surgeries using the data acquired from this literature review. The majority of anesthetic techniques utilized in Table 1 outlined the sole use of topical lidocaine to the corneal epithelium in cataract surgeries [5-6, 8-33]. However, it has been found that topical lidocaine gel, in conjunction with intracameral injection of lidocaine, can provide faster rates of visual rehabilitation post-surgery. Additionally, after analyzing the research articles, a common theme of using lidocaine concentrations of 2% was seen in Table 1. However, recent studies have recommended the strict clinical usage of lidocaine concentrations of only 1% to prevent endothelial cell damage and thickening of the cornea [7-8]. It is hoped that physicians will become aware of the contrast between current lidocaine practice and the ideal usage. However, it is also essential to keep in mind that these findings are standardized and the treatment of patients is variable to change per patient.

Further applications

Despite all of the current research that has been conducted on the application and usage of lidocaine on cataract surgery patients, additional studies are needed in order to determine more ideal standards that can be used for these procedures in order to minimize side effects that patients may experience. Currently, we understand that the standard concentration of lidocaine during cataract surgery is 2%; however, we do not have a mechanism or system to follow if this concentration needs to be adjusted in order to accommodate specific patient needs [7]. Supplementary research involving combination agents may be required in order to answer this question. Chemicals, such as ethylenediaminetetraacetic acid (EDTA), may be needed when conducting this research in order to test what effects different agents have on the analgesic effects of lidocaine [39-43]. In order to reduce the pain that patients may experience due to cataract surgery, appropriate usage of anesthetics is important. However, it is also quintessential that individuals with cataracts get an early diagnosis to prevent detrimental health complications and diminishing returns on surgery. Perhaps in the future, artificial intelligence (AI) software that analyzes retinal scans could be built to help increase the frequency at which patients with cataracts get diagnosed [44-49]. In order to protect the safety of cataract surgery patients, further research on the effects of lidocaine must be continued.

## Conclusions

Cataract surgery is a crucial medical procedure used to protect the eyesight of many individuals. Understanding the usage of anesthetics, such as lidocaine, during this procedure can help improve the current standard of patient care for this disease. Lidocaine is a sensory nerve dampener that can prevent pain by reducing stimulations from reaching the brain. Lidocaine is a commonly used anesthetic in cataract surgeries because it is efficient and requires little to no preoperative preparation. After analysis of the literature, it was found that the most commonly used combination agents with lidocaine in cataract surgeries were phenylephrine, tropicamide, tetracaine, povidone-iodine, and fentanyl. Additionally, a majority of physicians in the explored studies used lidocaine concentrations of 2% on the corneal epithelium. It is apparent that future improvements to lidocaine application in cataract surgeries need to be made. Contrary to the outlined practices, current studies have suggested that lidocaine is most beneficial when used both topically on the corneal epithelium and through an intracameral injection. It is hoped that the gap between current and ideal lidocaine practice in cataract surgery is shortened through this review.
